# Plant cell packs: a scalable platform for recombinant protein production and metabolic engineering

**DOI:** 10.1111/pbi.13081

**Published:** 2019-02-14

**Authors:** Thomas Rademacher, Markus Sack, Daniel Blessing, Rainer Fischer, Tanja Holland, Johannes Buyel

**Affiliations:** ^1^ Fraunhofer Institute for Molecular Biology and Applied Ecology IME Aachen Germany; ^2^ Institute for Molecular Biotechnology RWTH Aachen University Aachen Germany; ^3^ Present address: Pro‐SPR GmbH Schulstrasse 35 52477 Alsdorf Germany; ^4^ Present address: EPFL SV BMI LEN AI 2127 (Bâtiment AI), Station 19 CH‐1015 Lausanne Switzerland; ^5^ Present address: Indiana Biosciences Research Institute 1345 West 16th Street Indianapolis IN USA; ^6^ Present address: Eppendorf AG Bioprocess Center Rudolf‐Schulten‐Str. 5 52428 Juelich Germany

**Keywords:** high‐throughput screening, metabolic engineering, plant‐derived biopharmaceuticals, secondary metabolites, transient protein expression

## Abstract

Industrial plant biotechnology applications include the production of sustainable fuels, complex metabolites and recombinant proteins, but process development can be impaired by a lack of reliable and scalable screening methods. Here, we describe a rapid and versatile expression system which involves the infusion of *Agrobacterium tumefaciens* into three‐dimensional, porous plant cell aggregates deprived of cultivation medium, which we have termed plant cell packs (PCPs). This approach is compatible with different plant species such as *Nicotiana tabacum* BY2, *Nicotiana benthamiana* or *Daucus carota* and 10‐times more effective than transient expression in liquid plant cell culture. We found that the expression of several proteins was similar in PCPs and intact plants, for example, 47 and 55 mg/kg for antibody 2G12 expressed in BY2 PCPs and *N. tabacum* plants respectively. Additionally, the expression of specific enzymes can either increase the content of natural plant metabolites or be used to synthesize novel small molecules in the PCPs. The PCP method is currently scalable from a microtiter plate format suitable for high‐throughput screening to 150‐mL columns suitable for initial product preparation. It therefore combined the speed of transient expression in plants with the throughput of microbial screening systems. Plant cell packs therefore provide a convenient new platform for synthetic biology approaches, metabolic engineering and conventional recombinant protein expression techniques that require the multiplex analysis of several dozen up to hundreds of constructs for efficient product and process development.

## Introduction

Plant biotechnology has many potential applications, including the use of plants and plant cells to produce commodity or bulk chemicals such as biofuels and raw materials, through to specialty or fine chemicals including complex metabolites and recombinant proteins (Spiegel *et al*., 2018). The choice of expression host reflects a combination of host properties (food or feed crop, capacity for genetic manipulation, the presence of particular endogenous metabolic pathways, growth rate/productivity and intellectual property constraints) and product characteristics (intended purpose, structural complexity and degree of purification required) (Howat *et al*., 2014). A common goal is to optimize production in terms of yield, quality and purity, which typically involves the testing of different genes and gene combinations, or expression constructs containing various regulatory elements and targeting signals to control product accumulation (Bendandi *et al*., 2010; Buyel *et al*., 2013; Goojani *et al*., 2013).

Several transient expression platforms based on the infiltration of plants or plant tissues with *Agrobacterium tumefaciens* have recently been proposed for such screening purposes (Buyel and Fischer, 2012; Piotrzkowski *et al*., 2012). However, these systems are incompatible with typical microtiter plate formats, making the screening of multiple constructs or production conditions expensive and laborious. Furthermore, these systems are often prone to data variation due to subtle changes in cultivation conditions and biological heterogeneity that occur during the often long growth periods (40 days or more) required for whole plants. In contrast, plant cell suspension cultures can be grown under constant and defined conditions, allowing quality control particularly during continuous production processes (Sack *et al*., 2014). However, plant cells are not as scalable as whole plants, and processes that have been optimized for plant cell cultures are not always directly transferrable to plants, meaning that further screening and translational research are necessary.

To address this problem and create a bridge between the convenience of plant cells and the scalability of whole plants, we have developed a new screening platform based on plant cell cultures that are deprived of liquid medium to generate a plant cell pack (PCP). This is infused with *A. tumefaciens* to trigger the synthesis of recombinant proteins that are either the product (molecular farming) or enzymes that facilitate the production of particular metabolites (metabolic engineering). The method is compatible with microtiter plate formats, which allows the rapid, high‐throughput screening of different gene variants, expression constructs and process conditions individually or in combination. PCPs can also be cast in multi‐millilitre (up to ~150 mL) column or cake formats to facilitate the synthesis of products at the small preparative scale, for example, for initial functionality tests.

## Results and discussion

### PCPs outperform plant cells in terms of recombinant protein expression

**Figure 1:**
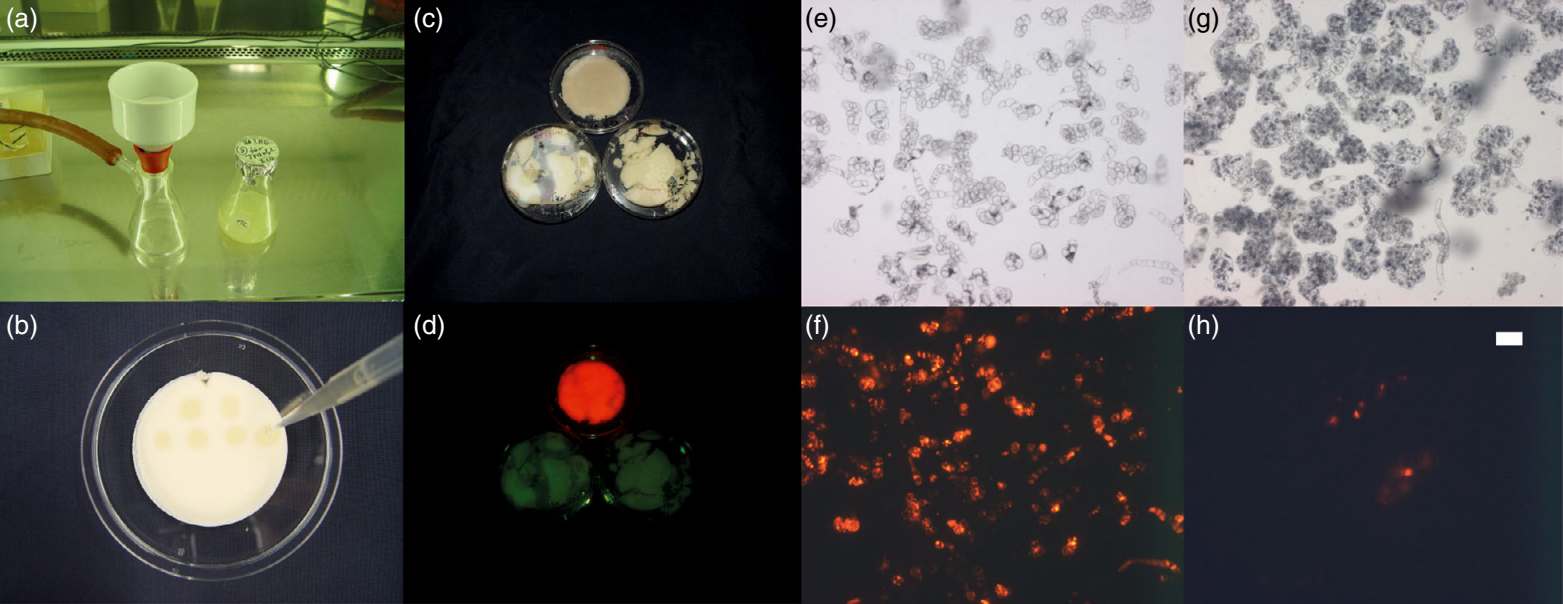
PCP generation, and DsRed expression in PCPs and BY2 cell suspension cultures. (a) Typical funnel filter used to prepare PCPs from plant cell suspension cultures. (b) *Agrobacterium tumefaciens* suspension applied to PCPs. (c) PCPs (top) and BY2 cells from suspension (bottom) after 5 days of DsRed expression under ambient light. (d) Same samples as in (c) but under green light, viewed through a red filter. (e) Microscopic image of resuspended cells from a PCP after 5 days of DsRed expression at 100‐fold magnification and under ambient light. (f) Same as (e) but using a filter cube with 545 ± 30 nm excitation and 610 ± 75 nm emission wavelength. (g) Microscopic image of BY2 cells from suspension after 5 days of DsRed expression under ambient light and 100‐fold magnification. (h) Same as (g) but using a filter cube as in (f). The size marker indicates 10 μm in panels (e–h).

Plant cell packs (PCPs) were cast by the vacuum filtration of a suspension of tobacco (*Nicotiana tabacum*) Bright Yellow 2 (BY2) cells through 0.2‐μm membranes to remove excess cultivation medium (Figure 1a). The resulting semi‐dry porous cell aggregates were transferred to a sterile support and infused by the dropwise addition of a suspension of recombinant *A. tumefaciens* carrying an expression vector for the target protein (Figure 1b). The porous structure of the PCP allowed the uptake of up to ~0.5 mL bacterial suspension per gram of PCP. For example, 50 mL of a BY2 cell suspension culture with a packed cell volume (PCV) of 30% [v/v] yielded a PCP weighing 4.5 g that we infused with 2.5 mL of *A. tumefaciens* suspension (OD_600nm_ = 1.0). In parallel, we used the same volume of the same BY2 culture and directly added the same volume of *A. tumefaciens* solution (without removing the cultivation medium) to compare the transfection efficiency of plant cells and PCPs based on the introduction of genes encoding the model fluorescent protein DsRed (Matz *et al*., 1999; Fradkov *et al*., 2000) and monoclonal antibody (mAb) 2G12 (Trkola *et al*., 1996). Macroscopic (Figure 1c,d) and microscopic analysis (Figure 1e–h) of DsRed fluorescence revealed hardly any fluorescence in BY2 cells that were transformed in suspension, whereas the PCPs displayed a distinct red colour. These results were confirmed when we compared the extracts from BY2 cell suspension cultures and PCPs. In the latter, the yields of DsRed and 2G12 were 55 and 47 mg/kg cell biomass, respectively, whereas both proteins were undetectable in BY2 cells transformed in suspension. The transfection efficiency in BY2 suspension cells was less than 5% (Figure 1h), whereas it was higher than 50% in the PCPs (Figure 1f). Certain properties of the PCPs, such as the higher cell density compared to cells in suspension, probably mimic the conditions found in leaves, increasing the likelihood of *A. tumefaciens* cells attaching to cells and transferring T‐DNA into them. The yields of DsRed and 2G12 in the PCPs were similar to those previously reported for transient expression in intact tobacco plants (103 and 55 mg/kg for DsRed and 2G12 respectively; Buyel and Fischer, 2012; Buyel *et al*., 2013). We therefore speculated that the expression data obtained from PCPs might correlate with and be translatable to intact plants, allowing PCPs to be used to screen for appropriate constructs and expression conditions.

### PCPs are similar to intact plants in terms of recombinant protein yields

**Figure 2:**
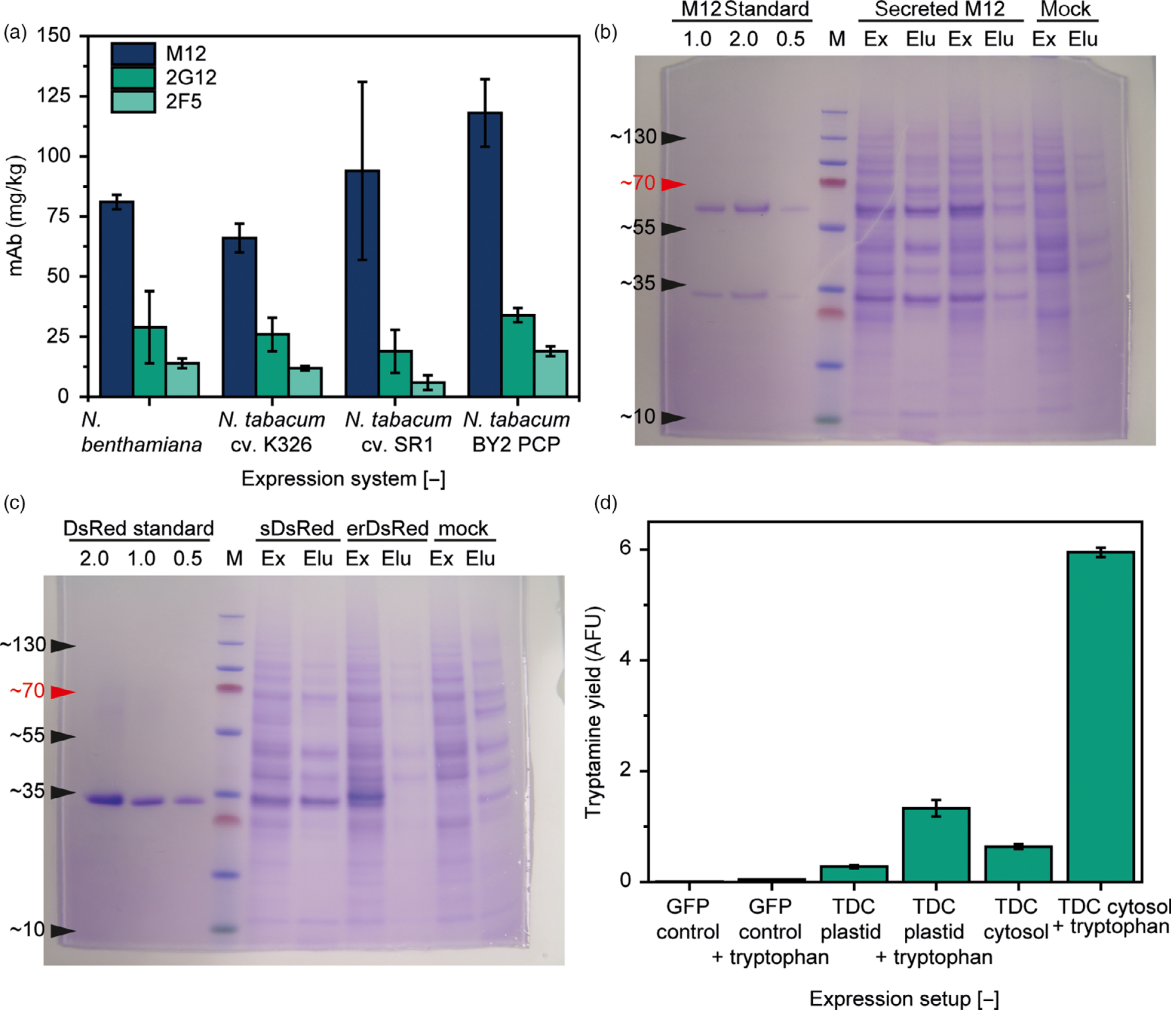
Protein expression in PCPs compared to intact plants. (a) Comparison of mAb expression levels in intact plants and PCPs 5 days after infiltration with *Agrobacterium tumefaciens*. (b) LDS polyacrylamide gel stained with Coomassie Brilliant Blue, showing extracts (Ex) and eluates (Elu) of PCPs after 5 days of expression of secreted mAb M12 or a mock control (infiltrated with untransformed bacteria). (c) LDS polyacrylamide gel stained with Coomassie Brilliant Blue, showing extracts (Ex) and eluates (Elu) of PCPs after 5 days of expression of secreted (sDsRed) or ER‐retained (erDsRed) DsRed as well as a mock control (infiltrated with untransformed bacteria). (d) PCPs expressing recombinant TDC or a GFP control were infused with 50 mm tryptophan (TDC substrate) 18 h post‐infiltration with *A. tumefaciens*. The accumulation of the product tryptamine was measured in PCP extracts 48 h later using a fluorescence assay (Sangwan *et al*., 1998). Error bars in (a) and (d) correspond to the standard deviation of *n* = 3 (intact plants and TDC expression) or *n* = 32 (mAb expression in PCPs).

We confirmed this assumption by expressing two other mAbs of the IgG class (M12 and 2F5; Muster *et al*., 1993; Wong *et al*., 2001) in PCPs, the tobacco varieties K326 and SR1 and in whole *N. benthamiana* leaves. Even though there were significant differences [two‐sided *t*‐test, 0.05 α‐level, *n* = 3 (intact plants) or *n* = 32 (plants vs. PCPs)] in the absolute expression levels, the qualitative results were the same for all expression systems (Figure 2a) 5 days after infiltration, a time at which the accumulation rates of recombinant proteins were found to be highest in the absence of silencing inhibitors (Buyel *et al*., 2013). Furthermore, the expression levels observed in the PCPs correlated strongly with those in *N. benthamiana* and *N. tabacum* cv. K326 as well as cv. SR1 (adjusted *R* ^2^ values of 0.99, 0.98 and 0.99 respectively; Figure S1a–c). Despite the high correlation coefficients, indicating a good match between the linear models and the data, there were cultivar‐specific differences such as different slopes when comparing whole plants and PCPs, perhaps reflecting physiological differences such as water content (Buyel *et al*., 2016), capacity for biosynthesis (Buyel and Fischer, 2012) or susceptibility to transfection by *A. tumefaciens* (Simmons *et al*., 2014). It will therefore be interesting to determine whether there are species‐dependent correlation factors between PCPs and certain plants that can facilitate a rapid extrapolation of expression screening results obtained in PCPs to intact plants.

On the other hand, expression levels were higher in PCPs than intact plants, suggesting that PCPs are advantageous as an expression platform at least for the three antibodies we compared. We did not use silencing inhibitors in our study, so transient co‐expression with p19 in *N. benthamiana* may result in higher product accumulation compared to the PCPs. However, the volumetric productivity of the PCPs is likely to be higher than in intact plants. For example, the packing density of PCPs was ~0.550 kg/L, whereas a density of ~0.750 kg/L has been reported for tobacco plants (Buyel *et al*., 2016) which typically occupy only ~0.5% [v/v] of the cultivation volume (e.g. a vertical farm) with their biomass, corresponding to an overall density of ~0.005 kg/L. Therefore, the volumetric productivity of plants based on the volume of the cultivation facility is only ~0.07 ± 0.03 mg/L/day (*n* = 3) for *N. tabacum* cv. SR1, whereas *N. tabacum* BY2 PCPs achieve ~12.98 ± 1.54 mg/L/day (*n* = 32) allowing for facilities with a much smaller footprint.

### Secreted proteins are easily recovered from PCPs

We further speculated that the artificial loose packing of cells in the PCPs and the absence of physical barriers (such as the cuticle and epidermis of intact leaves) would allow the rapid elution of secreted products. For example, infusing PCPs with buffer followed by vacuum filtration was sufficient to elute secreted recombinant proteins, which we demonstrated for both the mAb M12 and DsRed. Approximately 55% of the total M12 yield (175 mg/kg biomass) and 40% of the total DsRed yield (70 mg/kg biomass) were recovered by elution from the PCPs, as specifically determined by surface plasmon resonance spectroscopy (M12) and fluorescence analysis (DsRed). These recoveries were similar to those previously reported for centrifugal extraction from leaves (Kingsbury and McDonald, 2014; Turpen, 1999). If the same proteins were expressed in the cytosol, <1% of the total target protein was detected in the PCP eluates, indicating negligible release when the proteins were not secreted to the apoplast. These results were confirmed by LDS‐PAGE, which also indicated the minimal release of host cell proteins (Figure 2b,c). This is a useful attribute of PCPs because the retention of intracellular proteins also prevents the release of host cell proteins into the eluate, thus simplifying downstream processing, specifically product purification, by reducing the quantity of soluble impurities (Buyel and Fischer, 2014b). Product elution steps were applied several times to the same PCP, for example, to increase product recovery or to recover labile (e.g. pH sensitive) products before the final PCP harvest and thus prevent their denaturation or degradation.

### PCPs are also suitable for the production of specialized or secondary metabolites

In addition to recombinant protein expression, plant cells can be engineered to produce secondary metabolites for technical or pharmaceutical applications (Buyel, 2018; Staniek *et al*., 2013; Zhong, 2002). We expressed plastid‐targeted and cytosolic versions of the enzyme tryptophan decarboxylase (TDC; UniProtKB P17770) (Pennings *et al*., 1989) in PCPs to convert tryptophan into tryptamine, an early step in the biosynthesis pathway leading to therapeutically relevant alkaloids such as vinblastine and vincristine in *Catharanthus roseus*. PCPs transfected with TDC expression constructs were able to produce tryptamine (Figure 2d). The cytosolic construct yielded about twice as much tryptamine as its plastid‐targeted counterpart. Furthermore, by infusing the PCPs with 50 mm tryptophan 18 h post‐infiltration, we increased the tryptamine accumulation by a factor of 4.8 ± 0.1 (*n* = 3) for the plastid‐targeted TDC and by 9.3 ± 0.6 (*n* = 3) for the cytosolic TDC. The differences in yield between the cytosolic and plastid‐targeted TDC may reflect the abundance of the recombinant enzyme and/or the availability of the substrate in different subcellular compartments, for example, due to mass transport limitations in case of the vacuole where the precursor has to pass through more membranes. However, the native subcellular localization of TDC is the cytosol (Noe *et al*., 1984; Pennings *et al*., 1989), and the higher yields we observed for this construct are likely to reflect the adaptation of TDC to this compartment. The experiment established that PCPs are suitable for metabolic engineering experiments, for example, the screening of enzyme libraries, and that they also allow the feeding of substrates or intermediates.

### PCPs can be cast into various formats for screening and preparative applications

**Figure 3:**
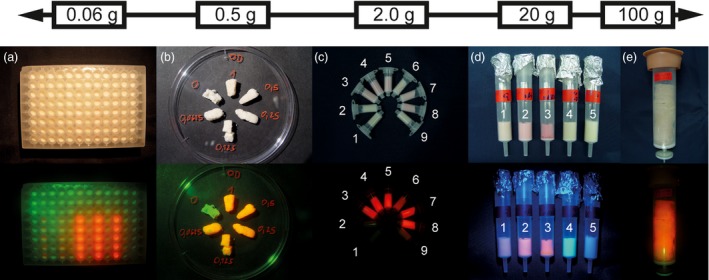
PCPs cast at different scales facilitate high sample throughput or initial product preparation. (a) PCPs expressing DsRed or a GFP control. (b) Sliced PCP fragments infused with *Agrobacterium tumefaciens* suspensions at different OD_600nm_ values. (c) PCPs cast into 2‐mL single‐use columns expressing (clockwise, starting left): (1) plastid‐targeted GFP, (2) untransformed cells, (3) secreted DsRed, (4) ER‐retained DsRed Munro and Pelham (1987), (5) cytosolic DsRed, (6) plastid‐targeted DsRed, (7) protein‐body‐targeted DsRed Geli *et al*., 1994, (8) co‐transformation with ER‐retained DsRed and plastid‐targeted GFP, (9) untransformed cells. (d) Expression of DsRed (2 – cytosolic, 3 – ER‐retained) or GFP (1 – cytosolic, 4 – plastid‐targeted) Prasher *et al*., (1992) in 20‐g PCPs cast into single‐use columns along with an untransformed control (5). (e) DsRed expression in a PCP composed of 100 g BY2 cells. Pictures were taken under ambient light conditions (top row) or under green light through a red filter (bottom row) to visualize DsRed fluorescence 5 days post‐infiltration with *A. tumefaciens*.

Having cast the PCPs described above using common Büchner funnels, we next set out to determine whether the cell aggregates were compatible with other formats for high‐throughput screening or preparative production (Figure 3).

First, we simply sliced the initial PCPs into pieces and used these for infusion with *A. tumefaciens* in separate containers, which reduced the chance of cross‐contamination and facilitated the analysis of incubation conditions such as different temperatures. Using 400 mL of BY2 cell culture with a PCV of 30%, we cast 20 PCPs, each facilitating the analysis of 200 samples, that is, different genes or gene variants. The method required only a few seconds for *A. tumefaciens* infusion, whereas injecting a bacterial suspension into leaf areas can take several minutes and the expression is influenced by the plant's age and physiological state. PCP infusion was more straightforward in terms of apparatus (only a small table‐top vacuum pump) and space requirements (~1 m lab bench) and did not require any special training (which is necessary for leaf injection). Furthermore, the macroscopic detection of DsRed was easier than in intact plants due to the near absence of autofluorescence in the BY2 cells used to cast the PCPs, reflecting the lack of chlorophyll in these cells (Figure 3b). The PCP slice format can therefore facilitate medium‐throughput screening with very low consumables costs, making the technique accessible to laboratories in developing countries. The slice format uses only ~0.5 g of BY2 cells per PCP and we intend to transfer the system to a smaller scale compatible with automated liquid handling to increase the throughput and multiplexing capability even further.

We were also able to replace the Büchner funnel with typical 96‐well microtiter plates yielding individual PCPs of ~60 mg that were infiltrated with *A. tumefaciens* and subsequently incubated *in situ* (Figure 3a and Figure S2a). Preparation of the plates and infusion with bacterial suspensions were achieved in less than 30 min, resulting in a throughput of several hundred samples per day per operator. In our hands, this has so far amounted to at least a fivefold increase in throughput compared to a recently reported leaf disc assay (Piotrzkowski *et al*., 2012) and reduced the laboratory space required from several square meters to only a few square centimetres. Handling steps such as pipetting and the removal of medium could be automated in the future, thereby further increasing the sample throughput and reducing inter‐sample variance. PCPs are therefore suitable for ambitious screening campaigns focusing for example on (i) multiple variants for a specific protein, (ii) the influence of multiple regulatory elements, (iii) the simultaneous expression of different combinations of transgenes and (iv) testing different sets of cell lines, for example, from tobacco, *N. benthamiana* and carrot (*Daucus carota*) (Figure S2a). Elucidating the reason why some of the carrot cell lines showed higher expression than others will require additional experiments.

Scale up from the PCP slices was also possible. For this purpose, we selected a column format that can support the elution of secreted products from the PCP as discussed above. We tested PCPs spanning four orders of magnitude using 0.5–100 g of cells in a column format based on single‐use laboratory equipment (Figure 3b–e). Macroscopic DsRed fluorescence was easily detected in all formats by illuminating the columns with green light followed by observation through a red filter foil. Fluorescence‐based quantitation of DsRed in PCP extracts showed that there was no significant difference in expression levels as we increased the scale (two‐sided *t*‐test, 0.05 α‐level, *n* = 12). Given expression levels of 124 ± 26 mg/kg (*n* = 4) and the maximum PCP mass of 0.10 kg used in this study, ~12 mg of product can rapidly be derived from a single PCP within 10 days from the onset of plant cell cultivation or 5 days after casting the PCP. Hence, PCPs can support the rapid and cost‐effective provision of milligrams of product suitable for initial functional analysis.

### Limitations and future developments

Humid but well‐aerated conditions were needed to ensure the viability of the cells in the PCP. The incomplete removal of excess liquids, such as culture medium or infiltration medium, caused the pale and translucent PCPs to turn opaque and brown within 24–48 h (Figure S2b,c) and reduced DsRed yields from ~120 to ~5 mg/kg or lower, depending on the severity of browning. Hence, the macroscopic appearance of a PCP can be used as rapid quality indicator for the success of the infiltration procedure and PCP handling in general. However, ensuring sufficient aeration becomes increasingly difficult with increasing column size due to heterogeneous cell packing, a problem reminiscent of the resin‐packing issues encountered during large‐scale chromatography. Therefore, we will test additional formats such as thick‐layer setups to achieve further scale up.

## Conclusion

In the past, a great deal of work and time was required to test different genes, gene variants or combinations of genes in order to synthesize the desired product, for example, a recombinant protein or metabolite. Our PCP technology is a simple new plant biotechnology platform that allows the rapid testing of recombinant protein expression to define optimal conditions, and the platform itself can be scaled up or the selected conditions can be transferred to whole plants. The system allows the parallel testing of several hundred genetic constructs per day in milligram‐sized PCPs. Even without silencing inhibitors, the expression levels of several recombinant proteins including antibodies and fluorescent proteins were in the 50–100 mg/kg range and thus similar to the expression in intact plants. Additionally, PCPs can also be directly scaled up to ~100 g of biomass to provide milligram quantities of product for functional tests. Protein expression in PCPs can be achieved using standard *A. tumefaciens* infiltration within 5 days, which either directly yields the desired recombinant product or endows the cells with the ability to synthesize specific metabolites. Therefore, the system is well suited for synthetic biology applications where the introduction of metabolic pathways, for example, to produce complex alkaloids and terpenoids, can be tested in cells from different plant species in parallel.

## Experimental procedures

### Plant cell cultures

Plant cells of all species were cultivated in the same liquid medium (30 g/L sucrose, 4.3 g/L Murashige and Skoog salts, 100 mg/L inositol, 1 mg/L thiamine, 0.2 mg/L 2,4‐dichlorophenoxyacetic acid, 200 mg/L potassium dihydrogen phosphate, pH 5.6) in the dark on a rotary shaker (180 rpm) at 26 °C. Cells were passaged weekly into fresh medium using inoculums of 15%, 4% or 50% (v/v) for *N*. *benthamiana* cells, tobacco BY2 cells and carrot cells respectively.

### Recombinant *Agrobacterium tumefaciens*

*Agrobacterium tumefaciens* strain GV3101:pMP90RK was used for infiltration. Cultures were initiated by inoculating 5.0 mL yeast extract broth (5.0 g/L beef extract, 1.0 g/L yeast extract, 5.0 g/L peptone, 0.5 g/L magnesium sulfate, pH 7.4, supplemented with 50 mg/L carbenicillin and 25 mg/L kanamycin) with 50 μL of a glycerol stock. The bacterial cultures were grown at 26 °C for 3 days to an OD_600nm_ of ~5. The bacteria were pelleted by centrifugation at 1000 *g* for 2 min and resuspended to the desired OD_600nm_ in infiltration medium (50 g/L sucrose, 2.0 g/L glucose, 0.5 g/L Ferty 2 Mega (Planta Düngemittel, Regenstauf, Germany), pH 5.3, supplemented with 200 μm acetosyringone). The bacterial suspension was incubated for 1–4 h at 22 °C before infiltration.

### Plant expression vectors

The pTRA vector, a derivative of pPAM (GenBank AY027531), was used for all expression experiments and contained the genes for DsRed and the various antibodies controlled by the double enhanced Cauliflower mosaic virus 35*S* promoter (Figure S3). Appropriate signal sequences or tags were used to direct the recombinant proteins to specific subcellular compartments.

### Preparation and transfection of PCPs and cell suspensions

All steps were carried out under sterile conditions using sterile equipment. The BY2 cell suspension culture was dispensed into vessels of the desired shape and size. Excess medium was removed by vacuum filtration (500 mbar for 1 min) to generate a porous, semi‐dry PCP. Flat discs were generated by pouring an aliquot (20–50 mL) of a 4 to 11‐day‐old BY2 suspension culture into a 75‐mL Büchner funnel equipped with a 5.5‐cm MN615 cellulose filter (Macherey‐Nagel, Düren, Germany) and applying a vacuum to remove the culture medium. The resulting PCP was placed in a Petri dish to measure its mass. PCPs in columns up to 2 mL or multiwell plates were cast using wide‐bore 1‐mL tips. The columns and the plates were either directly connected to a vacuum (micro columns) or placed in a NucleoVac 96 vacuum manifold (Macherey‐Nagel) for multiwell plates. For larger disposable chromatography columns (Macherey‐Nagel), the cell suspension was poured directly into the column which was connected to a vacuum.

Flat discs were infused by the dropwise addition of *A. tumefaciens* suspension uniformly onto the PCP surface (0.5 mL/g) with no additional liquid‐removal step. For PCPs in columns or plates, the entire PCP was infiltrated with an excess volume of the bacterial suspension (1 mL/g). After incubation for 30 min at 22 °C, surplus liquid was removed completely by vacuum filtration as described above. The infused PCPs were then incubated at a relative humidity of 90–95% for 3–7 days at 26 °C in the dark. For mAb expression experiments, the comparison involved *n* = 3 intact plants and *n* = 32 PCPs.

For the transfection of BY2 cells in suspension, 2.5 mL of the bacterial suspension was added to the cells and incubated on a rotary shaker (180 rpm) at 26 °C in the dark. The co‐incubation suspension was harvested by vacuum filtration after 5 days.

### Transient expression in intact plants

*Nicotiana benthamiana* and *N. tabacum* seeds were germinated on stone wool blocks (Cultilène, Rijen, the Netherlands) and were cultivated in a greenhouse at 25/22 °C day/night temperature with a 16‐h photoperiod (180 μmol/s/m^2^; λ = 400–700 nm) at 70% relative humidity. The plants were irrigated with a 0.1% (m/v) solution of Ferty 2 Mega (Kammlott GmbH, Erfurt, Germany) for 42 days and then vacuum infiltrated with *A. tumefaciens* at 5 kPa for 30 s.

The infiltrated plants were transferred into phytotrons and incubated for 5 days at 70% relative humidity and 25 °C with a 16‐h photoperiod, using six Osram cool white 36 W fluorescent tubes per 0.7 m^2^ (75 μmol/s/m^2^; λ = 400–700 nm).

### Extraction and analysis of target proteins

Soluble proteins were extracted from PCPs by homogenization, which was achieved by sonication at 40 W in a Bandelin Sonopuls device for 2 × 30 s with a 0.9‐s interval in two volumes (m/v) of extraction buffer (50 mm potassium phosphate, 500 mm sodium chloride, 10 mm sodium bisulfate, pH 7.5). The cell debris was pelleted by centrifugation (15 min, 13 000 *g*, 4 °C) and the supernatant was used for analysis. The same procedure was applied for plants transiently expressing mAbs 2G12, M12 or 2F5, but the sonication was replaced with 3 × 30 s in a Warring blender.

Secreted proteins were eluted from column‐format PCPs in extraction buffer at a ratio of 1.0 mL/g PCP. The infused PCP was incubated for 30 min at 22 °C, then the liquid was recovered by vacuum filtration and applied to the same cells. After three consecutive washing steps, the eluate was clarified by centrifugation (15 min, 13 000 *g*, 4 °C) and used for analysis.

For microscopic analysis, we used standard epifluorescence microscopes and dissecting microscopes with 545 ± 30 nm excitation and 610 ± 75 nm emission wavelength filter sets (Leica, Olympus, Wetzlar, Germany). To visualize DsRed fluorescence macroscopically, excitation was achieved using a cold light source with 1 m fibre optics and a green excitation filter (Schott, Mainz, Germany). The red fluorescence was observed and photographed through a red filter (light red 182; LEE filters, Andover, UK) with a digital camera (DSC‐F717; Sony, Minato, Japan).

DsRed was quantified by fluorescence spectroscopy as previously described (Buyel and Fischer, 2014a). DsRed fluorescence in the supernatants was measured using a Synergy HT plate reader fitted with 530/25 nm (excitation) and 590/35 nm (emission) filter sets. Antibody levels were measured by surface plasmon resonance spectroscopy using a BIACORE T200 instrument with protein A coupled to a CM5 sensorchip and a human IgG1/κ antibody as the standard (Holland *et al*., 2010).

### Lds‐page

We used pre‐cast lithium dodecyl sulfate polyacrylamide gels (Thermo Fisher Scientific, Waltham, Massachusetts) and stained proteins in the gels with Coomassie Brilliant Blue G250 as previously described (Arfi *et al*., 2016).

### Tryptophan feeding experiments and analysis of tryptamine levels

Plant cell packs in column format were infiltrated with 2 mL suspension of *A. tumefaciens* transformed with constructs for the expression of (i) a plastid‐targeted green fluorescent protein (GFP), (ii) a plastid‐targeted TDC or (iii) a cytosolic TDC. After incubation for 18 h (see above), PCPs were infiltrated with either 2 mL half‐concentrated infiltration medium or with 2 mL half‐concentrated infiltration medium supplemented with 50 mm tryptophan. After incubation for 30 min at 26 °C, the solutions were again completely removed by vacuum filtration and the PCPs were transferred back to the cultivation cabinet. Tryptamine was extracted from 250 mg samples and assayed as previously reported (Sangwan *et al*., 1998). Water‐soluble compounds were extracted from the samples by sonication in two volumes (v/m) of extraction buffer (50 mm sodium phosphate, 500 mm sodium chloride, 10 mm sodium bisulfate, pH 7.5). After adding 0.9 mL distilled water to 0.1 mL of the cleared extract, we added 2.0 mL 5.0 m sodium hydroxide and 3.5 mL ethyl acetate. The emulsion was mixed for 10 s and incubated at 4 °C for 16 h to allow phase separation. The upper organic phase was analysed using an Aminco Bowman AB2 luminescence spectrometer (Spectronic Instruments, Thermo‐Fisher Scientific). Tryptamine fluorescence was measured at 280 nm excitation and 350 nm emission wavelengths with 4‐nm slit width for excitation and emission light and the photomultiplier voltage set to 575 V.

## Authors’ contributions

T.R. invented the method, formulated hypotheses, designed and carried out experiments, analysed data, wrote the manuscript, M.S. designed experiments, analysed and interpreted data, wrote the manuscript, D.B. carried out experiments, T.H. carried out experiments, R.F. coordinated the project and interpreted data, J.B. coordinated the project, analysed and interpreted the data, wrote the manuscript. All authors discussed the results, commented on the manuscript and concur with its submission.

## Competing financial interests

The authors declare no competing financial interests.

## Supporting information


**Figure S1** Relative expression of monoclonal antibodies in different plant species and PCPs.
**Figure S2** DsRed expression in various column formats.
**Figure S3** Schematic maps of vector transfer DNAs (T‐DNAs) used for transient expression of recombinant proteins in plant cell packs.
