# Supplementary material for: Plant cell packs: a scalable platform for recombinant protein production and metabolic engineering
Source: Plant Biotechnol J. 2019 Feb 14;17(8):1560–6. doi: 10.1111/pbi.13081 (PMC6662111; doi:10.1111/pbi.13081)
Supplement: Supplementary file 1 — Figure S1 Relative expression of monoclonal antibodies in different plant species and PCPs. Figure S2 DsRed expression in various column formats. Figure S3 Schematic maps of vector transfer DNAs (T‐DNAs) used for transient expression of recombinant proteins in plant cell packs. [file PBI-17-1560-s001.docx]

**Supplementary materials to:**

**Plant cell packs: a scalable platform for recombinant protein production and metabolic engineering**

*Thomas Rademacher, Markus Sack, Daniel Blessing, Rainer Fischer, Tanja Holland, Johannes Buyel*

**Supplementary materials**


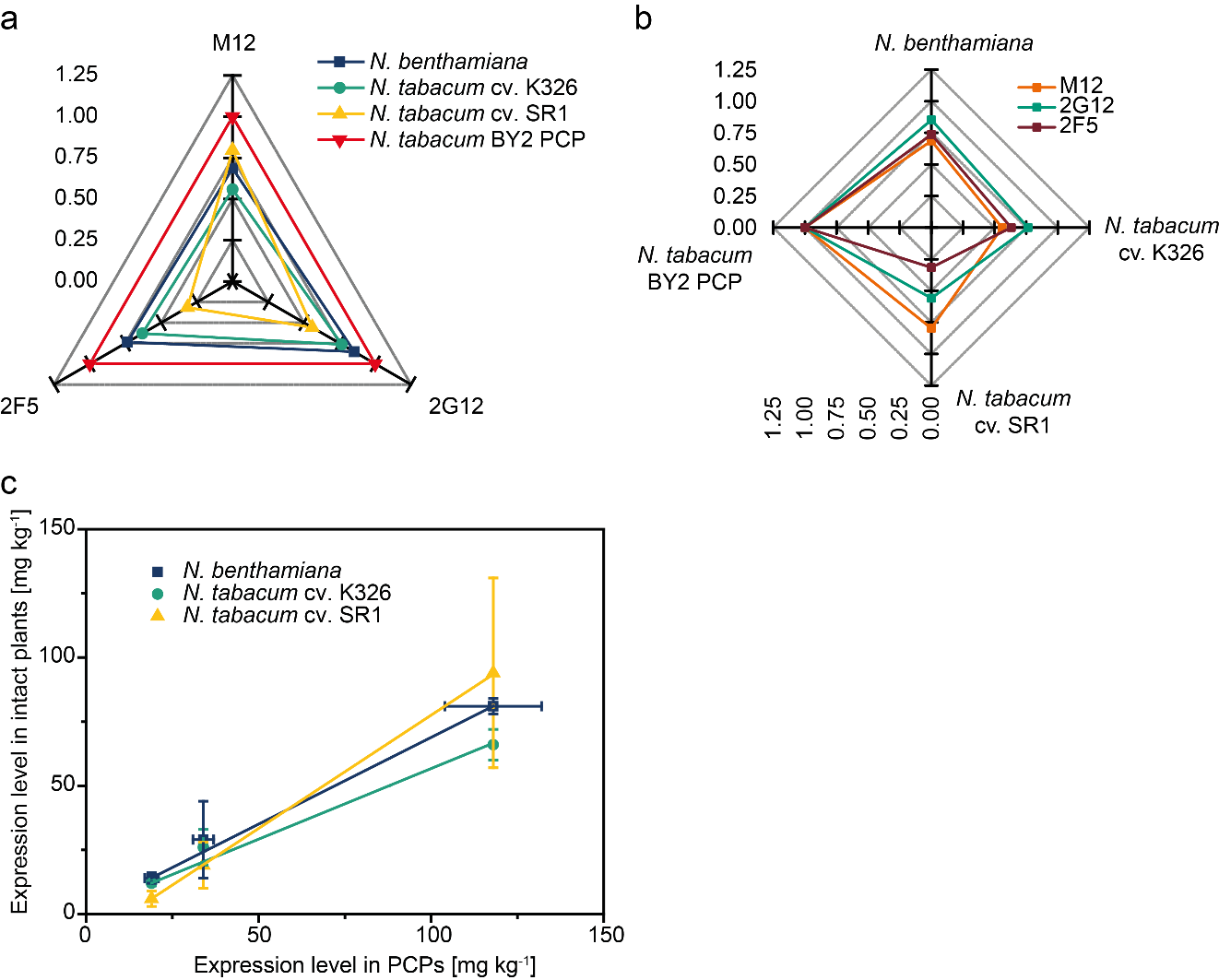


**Supplementary Fig. 1**. Relative expression of monoclonal antibodies in different plant species and PCPs. **(a)** Performance of *N. benthamiana*, two *N. tabacum* species and PCPs in expressing mAbs M12, 2G12 and 2F5, normalized to the highest expression observed (here, expression in PCPs). **(b)** Expression of mAbs M12, 2G12 and 2F5 according to the host system. Values are normalized to the maximal expression of each mAb, i.e. expression in PCPs. **(c)** Linear correlation between mAb expression in PCPs and plants. Vertical error bars indicate the standard deviation in plants (n=3) whereas horizontal error bars indicate the standard deviation in PCPs (n=32).


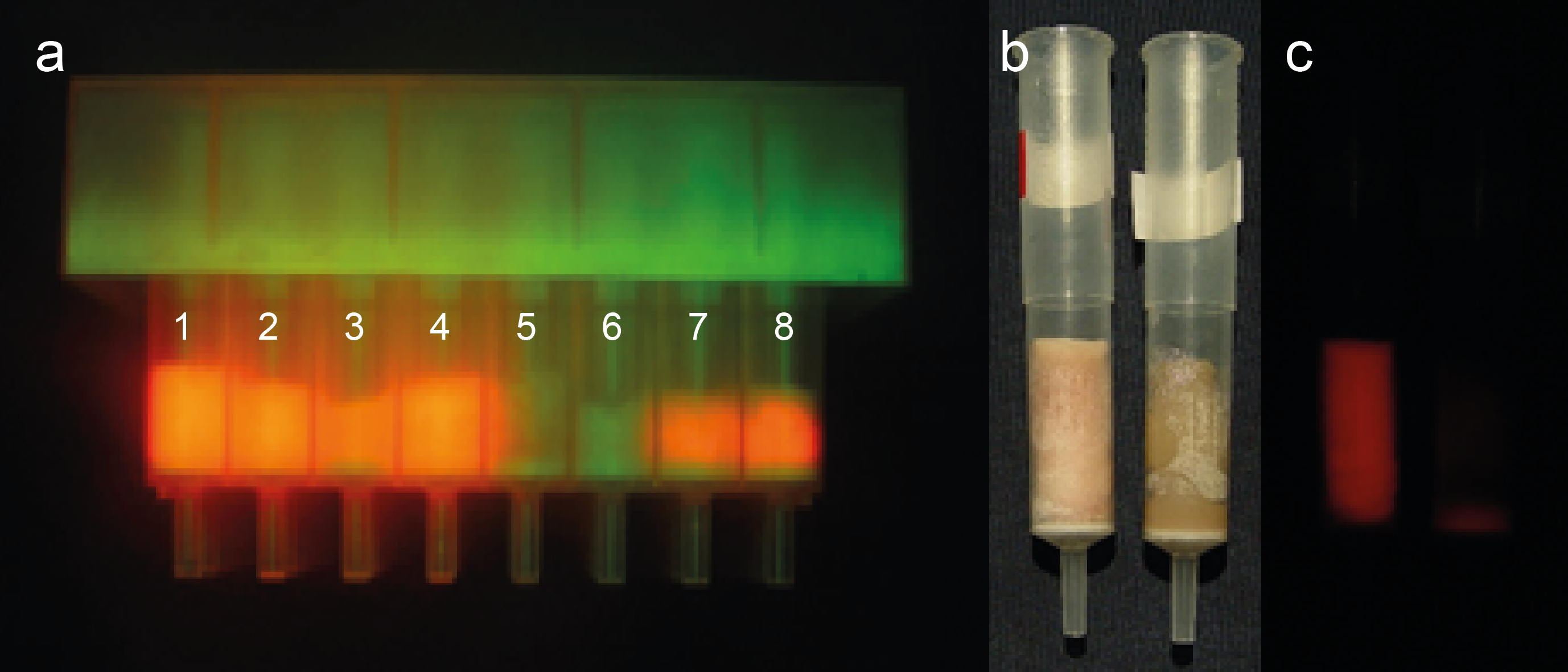


**Supplementary Fig. 2.** DsRed expression in various column formats. **(a)** PCPs cast from tobacco BY2 lines generated from different calli (1–3), from carrot lines PC-1106, PC-1164 and PC-1041 obtained from DSMZ (4, 5 and 6 respectively) as well as from *N. benthamiana* cells cultivated without (7) or with light (8) and infiltrated with *A. tumefaciens* at an OD_600nm_ of 0.1. The picture was taken through a red filter under green light after 5 days of expression of cytosolic DsRed. **(b)** PCPs in 20-g format that were fully (left) or incompletely (right) stripped of medium after infiltration. The picture was taken under ambient light after 5 days of expression of DsRed. **(c)** Same PCPs as in **(b)** but image taken through a red filter under green light.


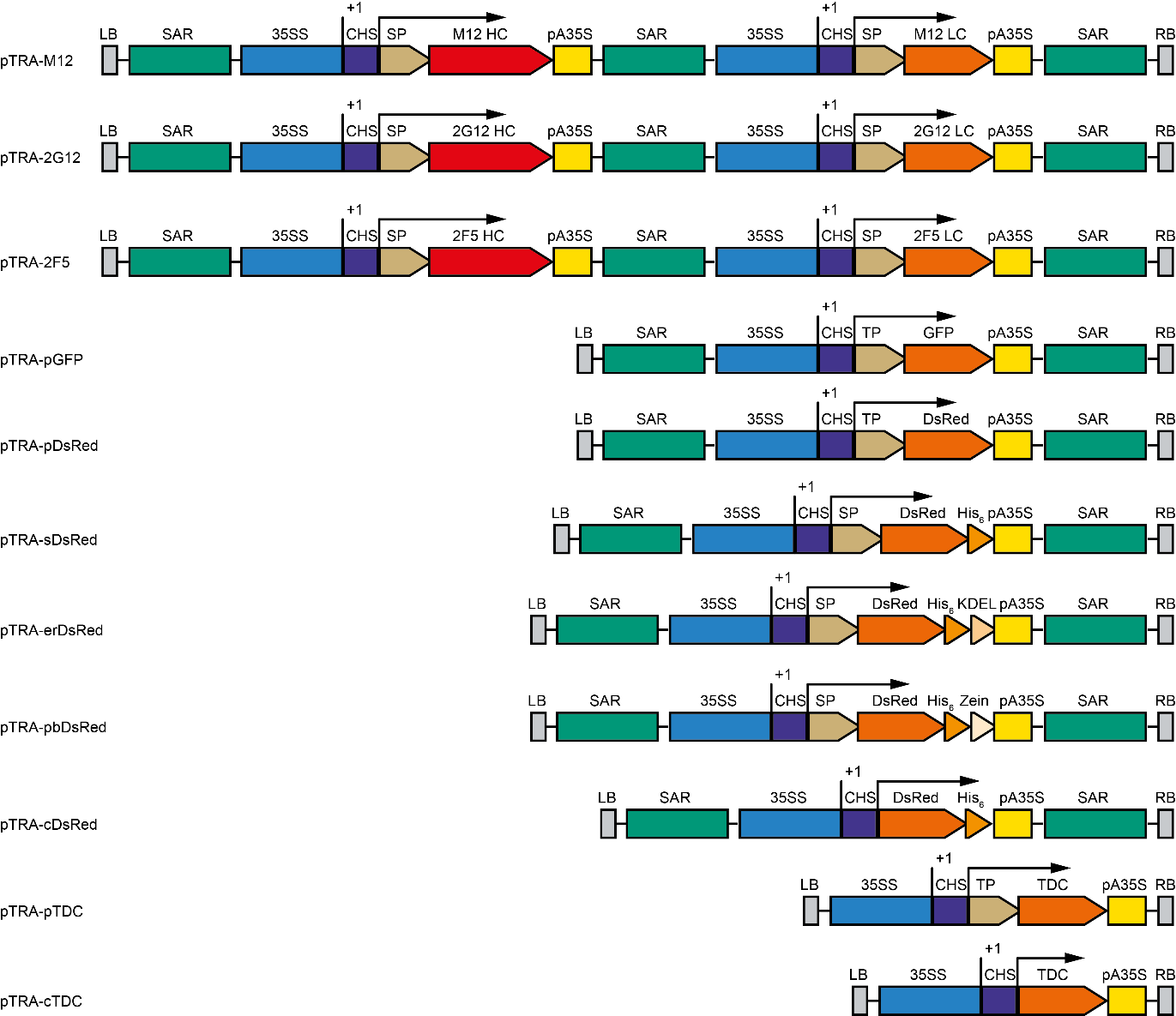


**Supplementary Fig. 3**. Schematic maps of vector transfer DNAs (T-DNAs) used for transient expression of recombinant proteins in plant cell packs. Vector pTRA, a derivative of pPAM (GenBank AY027531) was used as a backbone in all cases. Vectors from top to bottom: M12 – monoclonal antibody directed against cytosolic phospholipase A2 (Krarmer et al., 1991); 2G12 – monoclonal antibody directed against gp120 of HIV (Trkola et al., 1996); 2F5 – monoclonal antibody directed against gp41 of HIV (Muster et al., 1993); pGFP – plastid-targeted green fluorescent protein from *Aequorea victoria* (Prasher et al., 1992); pDsRed – plastid-targeted DsRed from *Discosoma* spp. (Fradkov et al., 2000); sDsRed – (secreted) apoplast-targeted DsRed; erDsRed – DsRed targeted to the endoplasmic reticulum (ER); pbDsRed – DsRed targeted to protein bodies using a zein-tag from *Zea mays* (Geli et al., 1994); cDsRed – cytosolic DsRed; pTDC – plastid-targeted tryptophan decarboxylase from *Catharanthus roseus* (Noe et al., 1984); cTDC – cytosolic TDC. Genetic elements: LB – T-DNA left border; RB – T-DNA right border; SAR – scaffold attachment region from the tobacco *RB7* gene (GenBank U67919); 35SS – double enhanced Cauliflower mosaic virus 35S promoter ; CHS – chalcone synthase 5′ untranslated region from *Petroselinum Hortense* (Kreuzaler et al., 1983); SP – signal peptide from murine monoclonal antibody mAb24 heavy chain (Fischer et al., 1999); HC – antibody heavy chain; LC – antibody light chain; pA35S – polyadenylation signal from Cauliflower mosaic virus; TP – transit peptide from *Solanum tuberosum* (GenBank X69762); His_6_ – hexa-His-tag; KDEL – SEKDEL (amino acid single letter code) tag for ER retention/retrieval (Munro and Pelham, 1987); Zein – zein-tag. The genetic elements are not drawn to scale.
